# Supplementary figures and images for: Expression of thermophilic two-domain laccase from Catenuloplanes japonicus in Escherichia coli and its activity against triarylmethane and azo dyes
Source: PeerJ. 2021 Jun 24;9:e11646. doi: 10.7717/peerj.11646 (PMC8236229; doi:10.7717/peerj.11646)

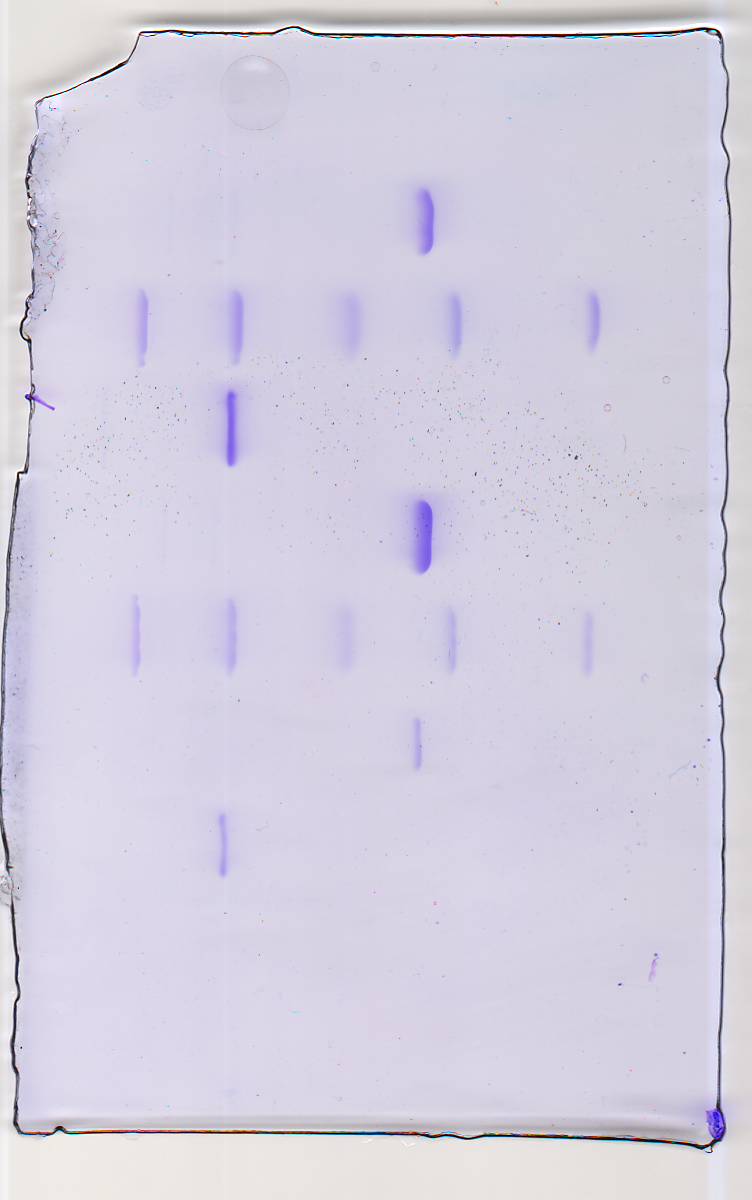

Supplement: Supplemental Information 3 [file peerj-09-11646-s003.zip › SDS-PAGE.JPG]

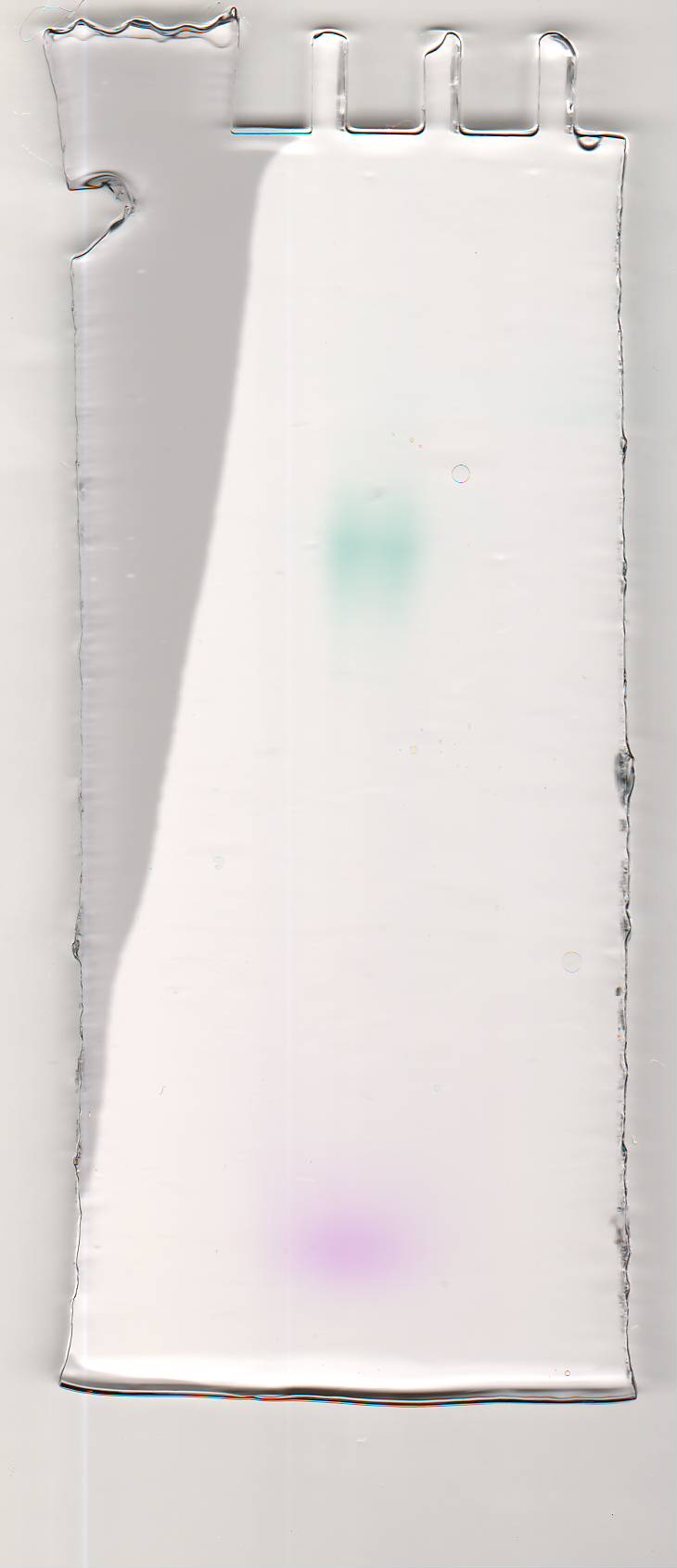

Supplement: Supplemental Information 3 [file peerj-09-11646-s003.zip › Native activity.JPG]

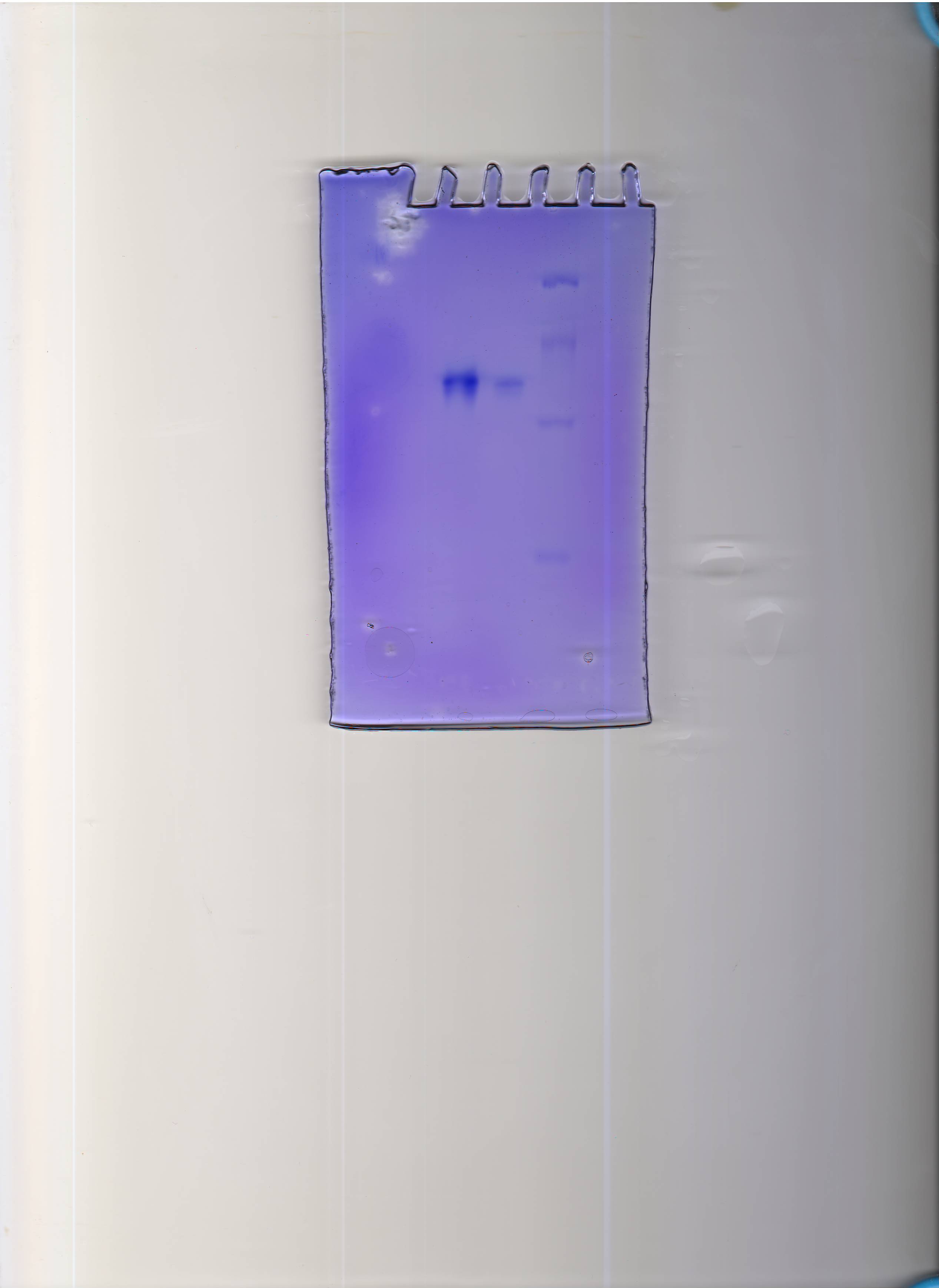

Supplement: Supplemental Information 3 [file peerj-09-11646-s003.zip › Native.JPG]
